# Supplementary material for: Behavioural evidence of a humidistat: a temperature-compensating mechanism of hydroregulation in spotted salamanders
Source: J Exp Biol. 2025 May 23;228(10):jeb250297. doi: 10.1242/jeb.250297 (PMC12148020; doi:10.1242/jeb.250297)
Supplement: Supplementary information [file jexbio-228-250297-s1.pdf]

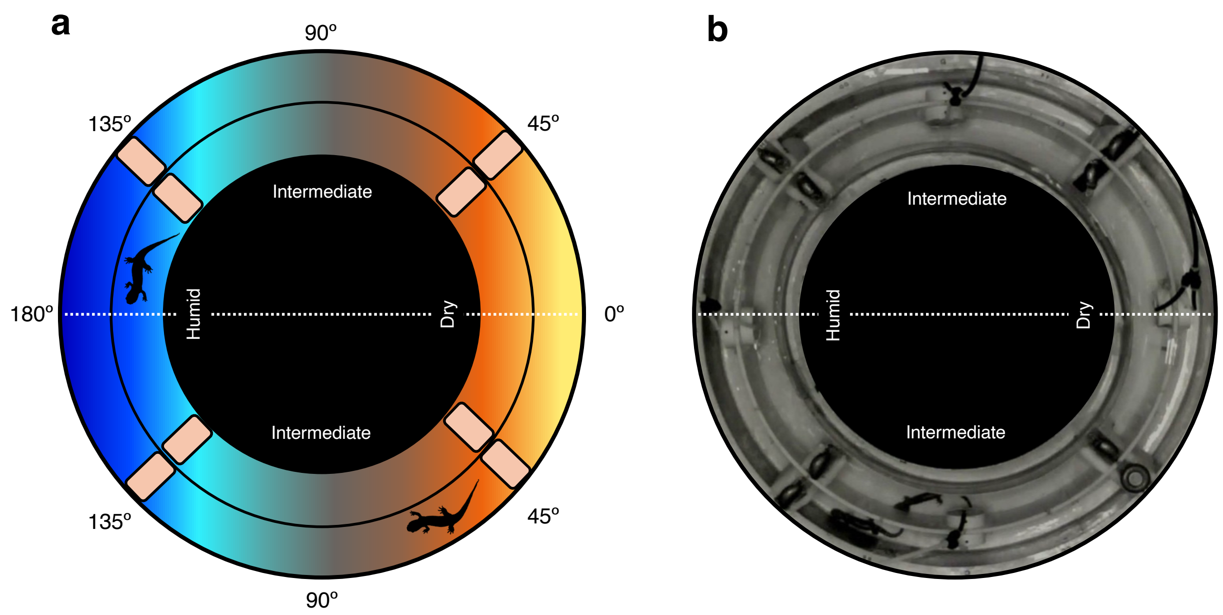

Fig. S1. a. Schematic of the annular humidity gradient used to assess how temperature affects behavioural hydroregulation in *Ambystoma maculatum*. The annular gradient is a mirror image along the horizontal axis. We affixed sponges at 45° and 135° to maintain humidity levels within gradient compartments. Gradient humidity is colour-coded, with colder colours indicating wetter conditions and warmer colours indicating dryer conditions. **b**. Actual image of the annular humidity gradient. The hygro-thermometers at the centre of each gradient compartment recorded temperature and relative humidity every 30 s during our 12 h long experiments. Two salamanders can be seen occupying the intermediate compartment on the bottom part of the humidity gradient.

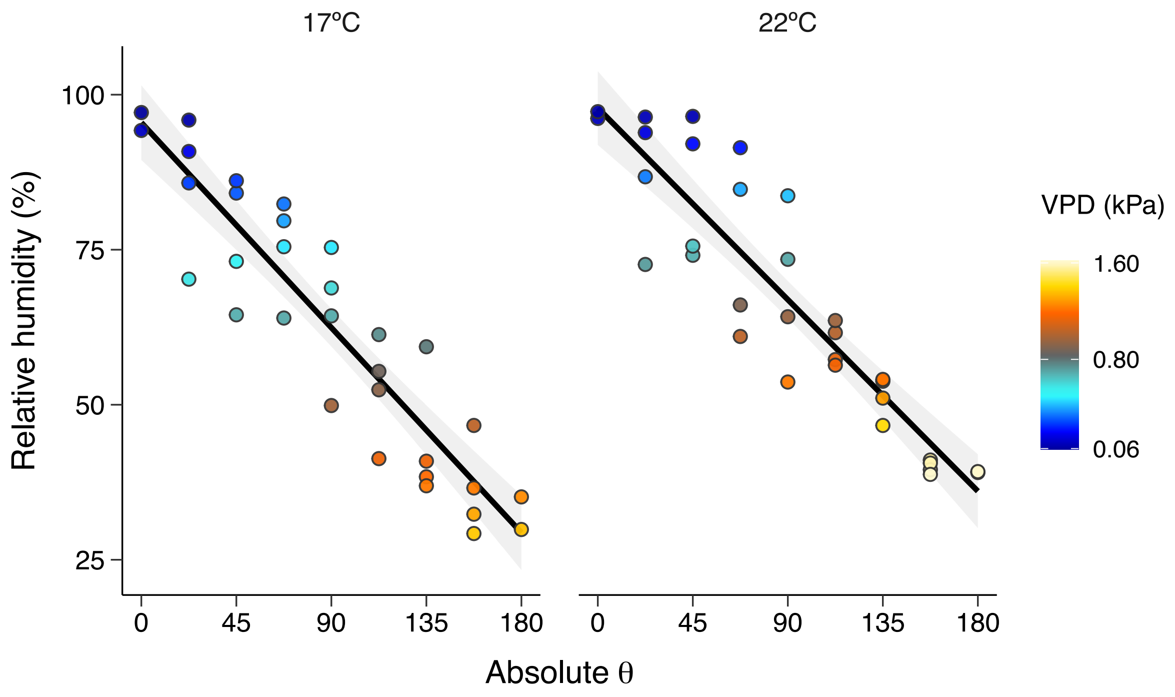

Fig. S2. Relationship between relative humidity and polar coordinates (absolute θ) of the annular gradient used in this study (kept either at 17°C or 22°C). Vapour pressure deficit (VPD) is colour-coded, with colder colours indicating wetter conditions and warmer colours indicating dryer conditions. At 17°C, the second-order regressions describing the relationship between relative humidity and gradient position followed: $y = -1_x10^{-4}x^2 - 0.35x + 94.85$ with $R^2 = 0.85$. At 22°C, the second-order regressions describing the relationship between relative humidity and gradient position followed: $y = -3_x10^{-4}x^2 - 0.29x + 96.20$ with $R^2 = 0.84$. The solid lines and shaded areas indicate the predicted relationship between relative humidity and absolute θ, and the 95% confidence interval, respectively.

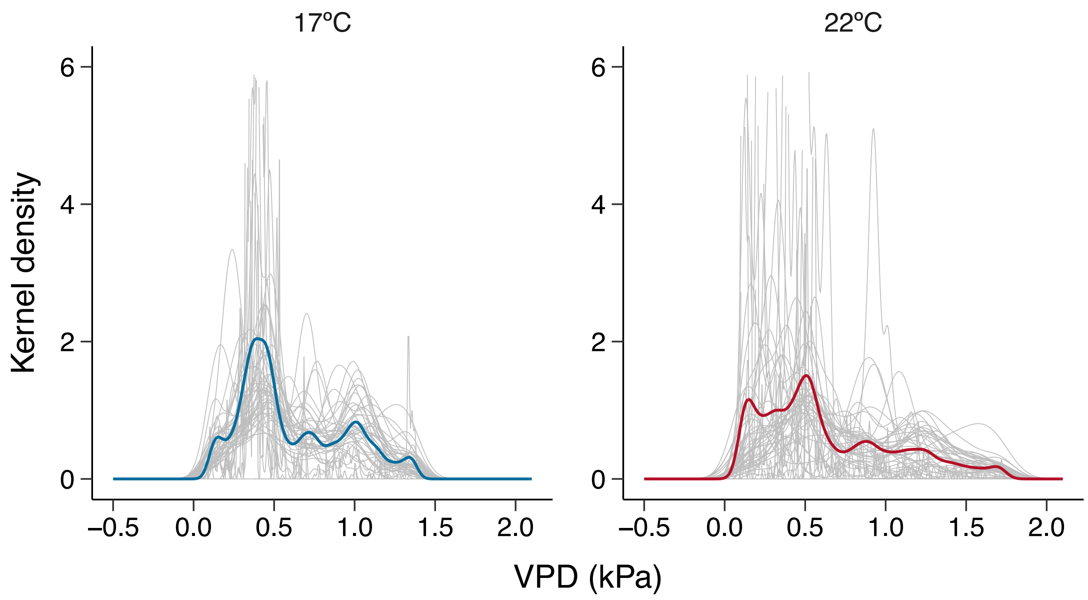

Fig. S3. Kernel density estimates of median selected VPD in *Ambystoma maculatum*. In both panels, grey lines show the individual distribution of VPD selection, and the colour-coded lines show the median VPD distribution considering all individuals tested at a given temperature. Values above 6 on the y-axis depict salamanders that selected a specific VPD for most of the experiment. The x-axis was expanded to negative values to include the tail of the kernel density estimates.

Journal of Experimental Biology • Supplementary information

Table S1. Experimental conditions of the humidity gradient used in the current study (kept at either 17°C or 22°C). We measured temperature and relative humidity (RH) every 60 seconds during a 24-h period to establish baseline gradient conditions in the absence of any animals. Below, humidity is expressed both as RH and as vapour pressure deficit (VPD).

	17°C			22°C		
	**Dry**	**Mid**	**Wet**	**Dry**	**Mid**	**Wet**
**Temperature (°C)**						
Mean ± SD	17.47 ± 0.35	16.93 ± 0.32	17.33 ± 0.28	22.17 ± 0.34	21.58 ± 0.21	22.12 ± 0.29
(min, max)	(16.42, 19.10)	(16.00, 18.47)	(16.80, 18.21)	(21.0, 22.52)	(21.10, 21.96)	(21.28, 22.39)
**RH (%)**						
Mean ± SD	37.56 ± 7.00	69.22 ± 15.09	93.41 ± 3.32	43.27 ± 5.61	68.50 ± 12.71	96.10 ± 2.04
(min, max)	(26.46, 74.70)	(45.81, 96.87)	(85.73, 99.30)	(36.22, 67.27)	(50.59, 96.24)	(89.67, 99.10)
**VPD (kPa)**						
Mean ± SD	1.25 ± 0.14	0.59 ± 0.297	0.13 ± 0.065	1.51 ± 0.16	0.81 ± 0.33	0.10 ± 0.05
(min, max)	(0.49, 1.50)	(0.06, 1.07)	(0.01, 0.30)	(0.81, 1.72)	(0.09, 1.30)	(0.02, 0.27)

SD = standard deviation, min = minimum value, max = maximum value.
